# Health-related quality of life and psychological distress in patients with brain tumors and their families: A cross-sectional web survey

**DOI:** 10.1093/noajnl/vdaf098

**Published:** 2025-05-18

**Authors:** Iori Sato, Mari Ikeda, Akemi Tsumura, Yoshitaka Narita

**Affiliations:** Department of Family Nursing, Division of Health Sciences and Nursing, Graduate School of Medicine, The University of Tokyo, Japan; Department of Family Nursing, Division of Health Sciences and Nursing, Graduate School of Medicine, The University of Tokyo, Japan; Yokohama Children’s Hospice Project, Kanagawa, Japan; Department of Neurosurgery and Neuro-oncology, National Cancer Center Hospital, Tokyo, Japan

**Keywords:** brain tumor, family, glioma, health-related quality of life, psychological distress

## Abstract

**Background:**

The diagnosis and treatment of brain tumors significantly impact not only patients but also their families. To develop better supportive care, it is essential to understand their quality of life (QOL) in detail.

**Methods:**

The Supportive Care for Brain Tumor Patients study group conducted a web survey targeting individuals aged 18 and above. Participants were recruited from a Japanese cohort and included patients and family members who responded to both the EQ-5D-5L (health-related QOL [HRQL]) and K6 (psychological distress) scales. Scores were compared with those of the general population, and multivariable regression analyses were performed.

**Results:**

A total of 136 patients and 103 family members were analyzed. Patients’ HRQL was lower than that of the general population. Family members’ psychological distress was higher than that of the general population. Factors associated with these quality-of-life indicators differed between patients and family members: decreased HRQL in patients was associated with financial hardship, being a homemaker, longer duration since diagnosis, and under treatment/relapse, while psychological distress in family members was associated with financial hardship and longer commuting time for hospital visits.

**Conclusions:**

The diagnosis and treatment of brain tumors significantly impact QOL for both patients and their families. In particular, support for the psychological well-being of family members is crucial, with emphasis on the importance of addressing lifestyle and caregiving needs.

**Key Points**

HRQL and psychological distress were good indicators of the family impact of the disease.
QOL was low in both patients with brain tumors and family members.
Nursing care of brain tumors requires assessment and support for the whole family.

**Importance of the Study**

With the shortening of hospitalization periods, the need for community care for individuals affected by brain tumors, including both patients and their families, is increasing. This study examined levels of HRQL and psychological distress in patients with brain tumors and their family members, and identified factors associated with these quality-of-life indicators. The findings revealed low levels of quality of life, especially in patients’ HRQL and family members’ psychological distress. Detailed characterization of the factors associated with these diminished outcomes provides valuable insights into when and how health and community specialists can effectively support the well-being of families of patients with brain tumors, namely financial support using a high-risk approach, mobility services for patients and family members, and whole-family assessment in consideration of the time-lag of disease impact on family.

The incidence rate of primary brain tumors is currently 23.9 per 100 000 people per year in Japan, and is increasing in line with the super-aged society.^1^ These tumors also occur in children, with an incidence rate of 2.26 to 2.63 per 100 000 children per year, making them the most common type of pediatric solid tumor. Brain tumors may be detected incidentally during health checkups or through symptoms such as headache and nausea. They exhibit diverse histological types, intracranial locations, and prognoses, and treatment is consequently integrative, involving a combination of surgery, radiation therapy, chemotherapy, immunotherapy, and other modalities. The onset, treatment, and sequelae of the disease significantly impact patients’ quality of life (QOL), necessitating long-term care and management. Although long-term care and management should involve family members, the QOL of patients and their family members is not sufficiently known.

The concept of QOL incorporates multiple aspects of people’s lives.^2^ The World Health Organization defines it as “an individual’s perception of their position in life in the context of the culture and value systems in which they live and in relation to their goals, expectations, standards, and concerns.” For individuals living with disease, injury, or impairment, the health-related aspects of QOL become particularly significant, as managing and coping with health challenges often occupy a substantial portion of their lives. Health-related QOL (HRQL) is defined as the health aspect of QOL that focuses on people’s level of ability, daily functioning, and ability to experience a fulfilling life.^2^ HRQL is a measurable, continuous concept which is influenced by a person’s objective assessment of their functional or health status, as well as their subjective perception of their personal health.^3^

Many researchers have investigated the HRQL of patients with brain tumors. Earlier work identified key determinants such as disease stage, treatment period, intracranial location, surgery, radiation, chemotherapy, supportive care, relapse, and low educational level.^4,5^ The HRQL of patients with brain tumors has been reported to be lower than that of healthy individuals and patients with other types of tumors, consistent with the type and period of disease and treatment.^5,6^ More recent systematic reviews have further clarified the HRQL challenges faced by patients with specific tumor types. For example, a review highlighted long-term HRQL impairments—including physical, cognitive, and social issues—among survivors of WHO grade II/III gliomas, often persisting for years after diagnosis and treatment.^7^ Similarly, Rimmer et al. found that adults with low-grade gliomas frequently experienced cognitive and emotional difficulties, fatigue, and long-standing functional impairments.^8^ These reviews emphasize the ongoing impact of brain tumors on patient functioning. While tumor-specific studies provide valuable insights, there is also a need for broader, tumor-agnostic approaches that examine HRQL across brain tumor populations. Such approaches can help identify high-risk subgroups—regardless of tumor type—who may benefit most from targeted support.

The family is an essential focus of nursing care. Not only patients themselves but also the family as a whole—including the health of individual family members, family relationships, and family functioning—are impacted by the disease, its treatment, and survivorship. The impact of disease on family caregivers has been assessed using several indicators, including burden, distress, HRQL, health condition, and absenteeism, among others.^9^ Among these, the HRQL of family caregivers of patients with brain tumors has been reported to be lower than population norms, particularly in cases of malignant brain tumors.^10^ Factors associated with the HRQL of family caregivers have been investigated not only in the contexts of brain tumors but also across a wide range of cancer populations, and include younger age, low income, and the patient’s mental or physical dysfunction.^11^ However, HRQL components and levels of psychological distress among caregivers of patients with brain tumors have been reported to be poorer and more distinct compared to those caring for patients with other types of cancer.^12^ Psychological distress has also been frequently observed among these caregivers.^13^ A recent systematic review by the RANO-Cares working group highlighted the accumulation of studies assessing psychological distress in this population, particularly those focused on anxiety or depression.^14^ More generally, Sun and colleagues reported that factors associated with high psychological distress included being female, poor caregiver health status, longer care time, younger caregiver age, caregiver not employed, and spouse.^15^ However, they did not investigate tumor-specific variables, such as duration after diagnosis or treatment modality. Identifying specific periods or subgroups of family members with particularly affected indicators is crucial to prioritizing them as targets for focused care. As with the patients themselves, understanding factors related to the psychological distress of families would be aided by a detailed understanding of factors related to their HRQL.

The purpose of our present study was to examine levels of HRQL and psychological distress in patients with brain tumors and their family members, and to identify the factors associated with these quality-of-life indicators. With the shortening of hospitalization periods, the need for community care for individuals affected by brain tumors is increasing, and includes both patients and their families.^16^ In Japan, while universal health coverage ensures access to specialized care across the country, cancer care centers are often concentrated in urban areas, placing additional burdens on patients and families living in rural regions. Furthermore, cultural expectations of familial caregiving remain strong, and many relatives—especially spouses or adult children—take on substantial care responsibilities, often outside of formal systems. Despite frameworks such as the Community-based Integrated Care System, informal caregiving continues to play a major role, potentially leading to hidden financial and emotional strain. Our goal was to provide insights into when and how health and community specialists can effectively support the well-being of families of patients with brain tumors.

## Methods

### Study Setting

The study was conducted as a secondary analysis of data from the “National Survey on the Needs and Support of Brain Tumor Patients and Caregivers.”^17^ The survey was conducted as a cross-sectional, web-based questionnaire between April and December 2023.

### Procedures

The survey was administered using Google Forms, an online questionnaire platform. Neuro-oncologists from several collaborating hospitals distributed the survey’s web link to patients and their families. Additionally, the web link was shared through various websites and social networking services that provide cancer-related information and support. Separate questionnaires were developed for patients and family members, and data from patients and family members within the same family could not be matched.

### Participants

The study included 2 distinct groups of participants: patients with brain tumors and their family members, including caregiving partners/acquaintances. Patients were eligible to participate if they were aged 18 years or older, had been diagnosed with or treated for a brain tumor, and could understand the research information, provide informed consent, and complete the questionnaire. Family members were eligible if they were aged 18 years or older, identified as a family member, partner, or acquaintance involved in the care of a patient with a brain tumor, and were similarly capable of understanding the research information, providing consent, and completing the questionnaire. Notably, family members of pediatric and adolescent patients were also included. Each patient and their family member participated independently, ensuring that their responses could not be matched to the same family unit.

### Ethical Considerations

This study was approved by the Research Ethics Review Committee of the National Cancer Center (Approval No.: 2022-430). Participants were first provided with detailed information about the study, and then indicated their understanding of the information and gave their consent to participate. All questions were optional, allowing participants to skip any question they did not wish to answer. The survey was designed to take approximately 15 to 30 minutes to complete. No financial compensation was provided to participants.

### Measurements

As a first quality-of-life indicator, the HRQL of patients and family members was measured using the Japanese version of the EuroQol 5-dimension instrument (EQ-5D),^18,19^ a self-reported, 5-item questionnaire consisting of “mobility,” “self-care,” “usual activities,” “pain/discomfort” and “anxiety/depression.” We used the 5-point Likert scale version (EQ-5D-5L) and transformed participants’ answers into a score of 0.000 to 1.000 based on Japanese value sets,^18^ with a higher score indicating higher HRQL. This scale has no clinically relevant cutoff points.

As a second quality-of-life indicator, the psychological distress of patients and family caregivers was measured using the Japanese version of the Kessler Psychological Distress Scale (K6),^20^ a self-reported, 6-item, 5-point Likert scale questionnaire. Participants were asked to describe the frequency with which they had experienced mood or anxiety symptoms over the past 30 days. Answers were summed to give a score of 0 to 24, with a higher score indicating higher distress. Although this scale has several clinically relevant cutoff points for different settings,^20^ we treated it as a continuous variable without applying cutoff points.

In the following, we describe the remaining background variables included in the analysis. This study was conducted as a secondary analysis, and therefore, not all ideal variables could be collected at the time of the original data collection. However, the authors were involved in planning the original survey and were able to include several relevant variables that were later used in this secondary analysis. Variable selection for analysis was not based on a specific theoretical model. However, in line with previous studies,^4,5,11,15^ we ensured the inclusion of variables commonly associated with HRQL and psychological distress, such as demographics, disease diagnosis, treatment stage, and treatment modality. In addition, based on discussions within the research team, we included other variables that were considered potentially relevant. We note, however, that objective income levels and cognitive function were not assessed in the original survey.

The survey also asked patients and family members about various other variables, including age, gender, degree of financial security, caregiving status, working status, and consultation usage. The question on financial security was adapted from the National Comprehensive Survey of Living Conditions^21^: “How do you feel about your overall current living situation?” Participants responded using a scale ranging from “High Financial Security” to “Considerable Financial Hardship.” The question on caregiving status first asked whether they were currently providing care to any family member(s), and if the answer was yes, then asked, “Who are you caring for?” The question about working status asked participants about their employment situation during the past week. Response options included: working, on leave, seeking work, student, homemaker, or not working. Regarding the last option, it was not possible to ascertain whether the reason for not engaging in work, study, or household responsibilities was due to illness, caregiver burden, or personal choice. The question about consultation usage asked participants whom they turn to for consultation, with the selection of multiple options allowed.

Patients also provided information on their residential prefecture; time since their brain tumor diagnosis; category (malignancy) and type of brain tumor; stage of tumor treatment; experience with surgical resection, radiation therapy, and chemotherapy; and commuting time for hospital visits. The brain tumor category (malignant or benign) and type were self-reported. Participants were asked to indicate whether they perceived their tumor as malignant, benign, or unknown. Responses were not mandatory to minimize the likelihood of inaccurate reporting. Family members reported the same information, but with specific regard to the patient rather than to their own details. Family members also reported their relationship to the patient and cohabitation status. The demographic questionnaire, originally developed in Japanese, has been translated into English and is provided in Supplementary Material 1.

### Historical Control

For EQ-5D-5L, we used data reported by Shiroiwa^18^ as control. This study reported scores from 1142 people in Japan aged in their 20s to 70s derived from research obtained during the development of the Japanese version of EQ-5D-5L. For K6, we used data reported by the National Comprehensive Survey of Living Conditions,^21^ including the K6 score distribution in Japan, derived from 80 612 males and females aged in their 20s to 70s.

Both studies had a sufficient sample size and reported scores for each age group (in 10-year intervals). Accordingly, when comparing HRQL/psychological distress in this study to these studies, we calculated age-adjusted scores standardized to match the age distribution of our present study rather than directly comparing the scores reported in these studies. We then compared these age-adjusted scores with the actual scores from this study.

### Statistical Analysis

All analyses were performed using R version 4.1.2,^22^ with statistical significance set at the 0.05 level. Consistent with the purpose of this study, cases missing either the EQ-5D-5L or K6 score were excluded from the analysis set. A priori sample size estimation was not conducted because this study was a secondary analysis which utilized the entire available sample.

To examine HRQL and psychological distress levels in patients with brain tumors and their family members, we conducted *t*-tests for 2 independent samples. Because the age distribution of patients and family members differed, patient and family member comparisons to their respective historical controls were conducted separately.

Factors associated with HRQL and psychological distress in patients with brain tumors and their family members were analyzed by multivariable regression analysis, with EQ-5D-5L or K6 as dependent variables. First, all potential independent variables were included in the regression model: patient age; gender; financial security; caregiving status; working status; time since brain tumor diagnosis; brain tumor category; treatment stage; experience with surgical resection, radiation therapy, and chemotherapy; and commuting time for hospital visits. The prefecture of residence was not included because a priori mixed-effects model analyses showed no random effect of residential prefecture in any model. Additionally, the type of brain tumor was excluded due to redundancy, and consultation usage was omitted because of causal reversibility concerns. Regression analyses of family members specifically included relationship to the patient and cohabitation status, whereas patient age and gender were excluded due to multicollinearity concerns. Missing answers and answers of “unknown” to any question were handled by multiple imputation using the R mice package. Second, to ensure that the results remained stable even when the set of independent variables was varied, we conducted multivariable regression analyses using not only the full model described above but also a trimmed model which excluded independent variables that were not significant.

Furthermore, we conducted 3 post hoc sensitivity analyses. First, because the number and range of patients resulted in the inclusion of various types of brain tumors, we were able to check the robustness of the results by multivariable regression analyses using the trimmed model among gliomas only (glioblastoma, grade 3 glioma, and grade 2 glioma). We hypothesized that results would be consistent when analysis was limited to gliomas only. Second, because the relationship between working status (which includes 6 categories) and patients’ HRQL was observed, we conducted an additional analysis by dichotomizing the working status into 2 categories: “working/student” and “not working.” This additional analysis aimed to clarify whether the association with HRQL was related to not working in general or to specific statuses, such as being a homemaker.

Third, in comparing results between patients and family members, we faced a limitation: because the data from patients and family members lacked shared identifiers (eg, familial IDs), it was not possible to directly match a patient’s data to that of a specific family member. Accordingly, comparisons could only be made between a group of patients and a group of family members from different families. To address this limitation, we conducted a post hoc sensitivity analysis using a probability-based matching approach. Specifically, we calculated Gower’s distance between all possible pairs of participants using variables such as patient age; gender; residential prefecture; time since brain tumor diagnosis; category and type of brain tumor; stage of tumor treatment; experience with surgical resection, radiation therapy, and chemotherapy; and commuting time for hospital visits. A patient and a family member were considered a matched pair only if they were each other’s closest match. We acknowledge that this method does not provide a validated or definitive way to identify true family pairs. Both patients and family members may make errors in self-reporting clinical or treatment information, and there may also be inconsistencies even within the same household. To mitigate this, our approach did not require exact matches but relied on similarity across multiple background characteristics.

## Results

### Participant Characteristics

The original study included data from 177 patients and 132 family members. In the present study, 41 patients and 28 family members were excluded due to missing EQ-5D-5L and K6 scores, leaving 136 patients and 103 family members for analysis.

Most patients were male (*n* = 77, 57.5%), working (*n* = 87, 64.0%), and diagnosed with malignant brain tumors (*n* = 110, 85.3%; Table 1). They represented various treatment stages and had undergone a range of treatments. The majority of patients sought consultation from family or relatives when needed (*n* = 132, 97.1%).

**Table 1.** Participant Characteristics

	Patient reports (*N* = 136)		Family member reports (*N* = 103)			
	Patient characteristics		Patient characteristics		Family member characteristics	
	*n*	%	*n*	%	*n*	%
Age						
00s	—	—	2	1.9	—	—
10s	—	—	5	4.9	—	—
20s	8	5.9	14	13.6	1	1.0
30s	21	15.6	4	3.9	11	10.9
40s	42	31.1	15	14.6	21	20.8
50s	37	27.4	16	15.5	33	32.7
60s	20	14.8	28	27.2	22	21.8
70s	7	5.2	15	14.6	13	12.9
80s	—	—	4	3.9	—	—
Gender						
Male	77	57.5	64	62.7	32	31.1
Female	57	42.5	38	37.3	71	68.9
Residential area						
Kanto or further north	95	72.0	67	65.7	—	—
Chubu or further west	37	28.0	35	34.3	—	—
Financial security						
High financial security	8	5.9	—	—	1	1.0
Moderate financial security	32	23.7	—	—	12	11.9
Average financial security	64	47.4	—	—	47	46.5
Mild financial hardship	28	20.7	—	—	31	30.7
Considerable financial hardship	3	2.2	—	—	10	9.9
Relationship to the patient						
Spouse/partner	—	—	—	—	65	63.1
Parent	—	—	—	—	21	20.4
Child	—	—	—	—	12	11.7
Sibling	—	—	—	—	5	4.9
Cohabiting status						
Living with the patient	—	—	—	—	88	85.4
Not living with the patient	—	—	—	—	15	14.6
Caregiving status						
No	119	89.5	—	—	67	65.0
Yes	14	10.5	—	—	36	35.0
(If Yes,) Who are you caring for?						
Infant or toddlers	9	—	—	—	2	—
Older adults	3	—	—	—	12	—
Individuals with disabilities or other conditions	4	—	—	—	24	—
Working status						
Working	87	64.0	—	—	60	58.3
On leave	17	12.5	—	—	4	3.9
Unemployed and seeking work	1	0.7	—	—	1	1.0
Student	2	1.5	—	—	—	—
Homemaker	10	7.4	—	—	26	25.2
Not working	19	14.0	—	—	12	11.7
Consultation with others (multiple answers allowed)						
Family/relatives	132	97.1	—	—	88	85.4
Peers affected by brain tumors	11	8.1	—	—	4	3.9
Acquaintances or friends	42	30.9	—	—	28	27.2
Medical or welfare professionals	28	20.6	—	—	25	24.3
Public consultation services	4	2.9	—	—	4	3.9
Private organizations (eg, patient groups)	4	2.9	—	—	1	1.0
Time since diagnosis of brain tumor						
<6 mo	13	9.6	12	11.7	—	—
6 mo to 1 yr	9	6.6	14	13.6	—	—
1 to 2 yr	18	13.2	17	16.5	—	—
2 to 5 yr	43	31.6	30	29.1	—	—
5 to 10 yr	28	20.6	18	17.5	—	—
>10 yr	25	18.4	12	11.7	—	—
Category of brain tumor						
Benign	19	14.7	2	2.0	—	—
Malignant	110	85.3	98	98.0	—	—
Type of brain tumor						
Metastatic brain tumor	9	7.2	5	5.0	—	—
Malignant lymphoma	4	3.2	5	5.0	—	—
Glioblastoma	34	27.2	41	40.6	—	—
Grade 3 glioma	26	20.8	21	20.8	—	—
Grade 2 glioma	32	25.6	10	9.9	—	—
Pituitary tumor	2	1.6	—	—	—	—
Schwannoma	2	1.6	2	2.0	—	—
Meningioma	7	5.6	2	2.0	—	—
Other benign brain tumors	4	3.2	3	3.0	—	—
Other malignant brain tumors	5	4.0	12	11.9	—	—
Stage of treatment						
Cured (under observation only)	43	33.1	23	23.7	—	—
Stable (under observation only)	45	34.6	26	26.8	—	—
Stable (under treatment)	29	22.3	28	28.9	—	—
Recurrence/progression	13	10.0	20	20.6	—	—
Experience with surgical resection of brain tumor						
Experienced	122	89.7	94	91.3	—	—
Not experienced	14	10.3	9	8.7	—	—
Experience with radiation treatment of brain tumor						
Past experience	82	60.3	83	81.4	—	—
Undergoing	6	4.4	2	2.0	—	—
Not experienced	48	35.3	17	16.7	—	—
Experience with chemotherapy for brain tumor						
Past experience	62	45.9	48	47.1	—	—
Undergoing	29	21.5	39	37.9	—	—
Not experienced	44	32.6	15	14.6	—	—
Commuting time for hospital visits						
<30 min	23	17.3	27	26.2	—	—
30 to 60 min	47	35.3	27	26.2	—	—
1 to 2 h	49	36.8	39	37.9	—	—
>2 h	14	10.5	10	9.7	—	—

Missing answers and answers of “unknown” were excluded.

Most family members were female (*n* = 71, 68.9%), working (*n* = 60, 58.3%), and the spouse/partner of a patient with a brain tumor (*n* = 65, 63.1%). Their family members with brain tumors were predominantly male (*n* = 64, 62.7%) and had been diagnosed with malignant brain tumors (*n* = 98, 98.0%), similar to the patients included in the study. Like the patients, most family members sought consultation from family or relatives when needed (*n* = 88, 85.4%).

### Levels of HRQL and Psychological Distress

**Figure 1.**
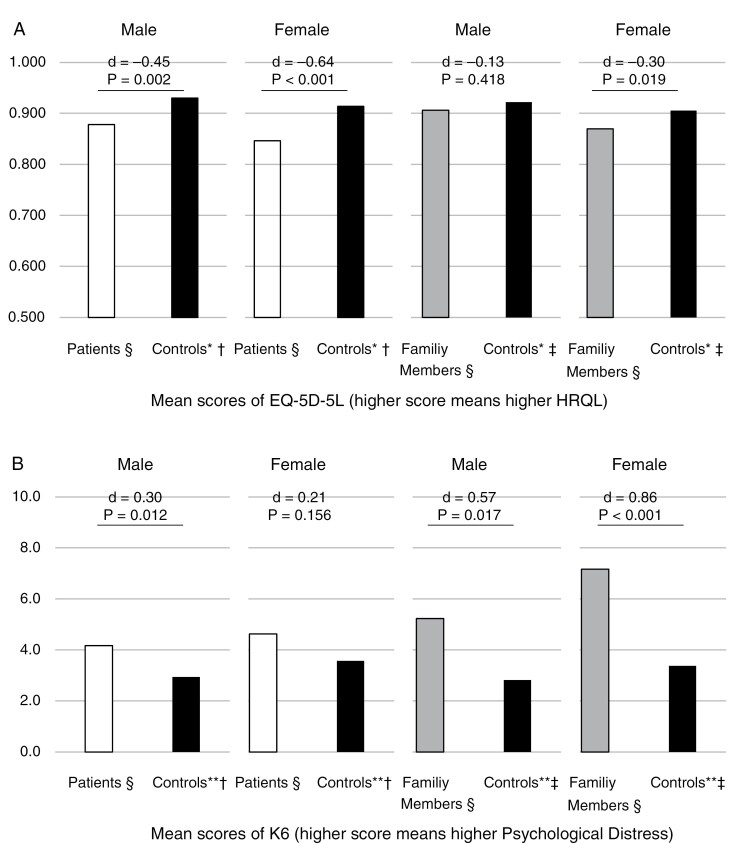
Health-related quality of life and psychological distress among patients with brain tumors and their family members. HRQL, health-related quality of life; K6, Kessler Psychological Distress Scale. * Data from Shiroiwa,^18^ which reported mean scores of EQ-5D-5L by age (20s to 70s or over) and gender (total *N* = 1142). ** Data from the Comprehensive Survey of Living Conditions,^21^ which reported K6 score distribution by age (5-year strata) and gender (total *N* = 80 612). † Standardized to match the age distribution of patients in this study for comparability. ‡ Standardized to match the age distribution of family members in this study for comparability. § Participants of this study, excluding nonrespondents to questions on age or gender.

Mean EQ-5D-5L score for patients was 0.863 (SD 0.134), with a median of 0.867 (interquartile range [IQR]: 0.769–1.000). Among both genders, the mean EQ-5D-5L scores for patients were lower than those of the age-adjusted general population (Figure 1) . The mean K6 score for patients was 4.5 (SD 4.8), with a median of 3.0 (IQR: 0.0–7.0). Among both genders, the mean K6 scores for patients were higher than those of the age-adjusted general population, with the standardized differences smaller than those for EQ-5D-5L, especially among females. The EQ-5D-5L score and the K6 score among patients were significantly correlated (Pearson’s *r* = −0.57; Spearman’s rho = −0.59).

The mean EQ-5D-5L score for family members was 0.879 (SD 0.124), with a median of 0.867 (IQR: 0.823–1.000). Among both genders, the mean EQ-5D-5L scores for family members were lower than those of the age-adjusted general population. The mean K6 score for family members was 6.5 (SD 5.9), with a median of 5.0 (IQR 1.5–11.0). Among both genders, the mean K6 scores for family members were higher than those of the age-adjusted general population, with standardized differences significantly larger than those for EQ-5D-5L, especially among females. The EQ-5D-5L score and the K6 score among family members were significantly correlated (Pearson’s *r* = −0.57; Spearman’s rho = −0.56).

For comparison between patients and family members, a matched subsample (*n* = 39) was extracted. Within this subsample, the EQ-5D-5L score for patients was 0.834 (SD 0.146), which was lower than that of family members (0.877, SD 0.888), although the difference was not statistically significant (d = −0.31, *P* = .064). Similarly, the mean K6 score for patients was 5.2 (SD 5.2), which was lower than that of family members (6.6, SD 5.5), but this difference was also not statistically significant (d = −0.24, *P* = .139).

### Factors Associated With HRQL and Psychological Distress

In patients, multivariable regression analysis revealed that low EQ-5D-5L scores were associated with financial hardship, being a homemaker, a long duration since diagnosis (>10 years), and being under treatment or experiencing tumor recurrence/progression (Table 2). The results of the full and trimmed models were closely similar, indicating that the findings were robust (Supplementary Material 2-1). The multivariable analysis among glioma patients only also indicated robustness. In the analysis using the dichotomized working status, it appeared that being a homemaker was more strongly associated with lower HRQL than simply being categorized as “not working.” High K6 scores in patients were associated with financial hardship only, with no other variable showing a statistically significant association.

**Table 2.** Factors Associated with Health-Related Quality of Life and Psychological Distress among Patients with Brain Tumors (N = 136)

	EQ-5D-5L					K6				
	Discription^*^			Full model^***^		Discription^*^			Full model^***^	
	Mean	SD	*P* ^**^	B	*P*	Mean	SD	*P* ^**^	B	*P*
Intercept				0.91	.000				0.0	.995
Age										
20s	0.89	0.153	.132	Reference		3.6	3.34	.144	Reference	
30s	0.92	0.080	.463	0.11	.066	2.8	3.74	.408	0.9	.708
40s	0.83	0.140		0.04	.437	5.8	5.15		2.6	.261
50s	0.85	0.135		0.05	.356	3.8	4.38		1.0	.677
60s	0.89	0.137		0.08	.192	4.1	4.71		2.5	.332
70s	0.85	0.136		0.05	.479	6.7	7.30		3.9	.181
Gender										
Male	0.88	0.130	.177	0.01	.558	4.1	4.35	.551	−0.4	.690
Female	0.85	0.136		Reference		4.6	5.10		Reference	
Residential area										
Kanto or Further North	0.88	0.130	.202			4.3	4.60	.758		
Chubu or Further West	0.84	0.135				4.6	5.09			
Financial security										
High financial security	0.97	0.093	<.001	0.08	.096	0.4	0.74	<.001	−1.5	.426
Moderate financial security	0.90	0.110	<.001	−0.02	.558	3.6	3.64	<.001	0.3	.756
Average financial security	0.88	0.121		Reference		3.7	4.47		Reference	
Mild financial hardship	0.76	0.125		−0.12	<.001	7.8	5.43		4.5	<.001
Considerable financial hardship	0.64	0.134		−0.19	.010	10.7	3.06		4.8	.109
Caregiving status										
No	0.87	0.130	.653	Reference		4.5	4.67	.931	Reference	
Yes	0.85	0.136		−0.01	.689	4.6	5.98		−0.5	.745
Working status										
Working	0.90	0.116	<.001	Reference		3.9	4.63	.199	Reference	
On leave	0.77	0.135		−0.05	.234	6.9	5.27		2.7	.105
Unemployed and seeking work	1.00	NA		0.33	.014	7.0	NA		−0.2	.970
Student	1.00	0.000		0.15	.128	4.0	5.66		3.0	.472
Homemaker	0.81	0.134		−0.11	.022	3.7	4.19		0.0	.985
Not working	0.81	0.153		−0.05	.143	5.5	5.10		0.2	.868
Consultation with others (multiple answers allowed)										
Family/Relatives	0.86	0.134	.738			4.4	4.74	.731		
Peers affected by brain tumors	0.82	0.130	.225			5.6	6.15	.368		
Acquaintances or friends	0.88	0.126	.233			3.7	4.16	.220		
Medical or welfare professionals	0.83	0.127	.096			4.8	5.14	.634		
Public consultation services	0.72	0.100	.036			7.3	4.92	.221		
Private organizations (eg, patient groups)	0.73	0.095	.042			5.5	3.87	.639		
Time since diagnosis of brain tumor										
<6 mo	0.83	0.109	.741	0.05	.324	4.9	4.81	.940	−1.0	.624
6 mo to 1 yr	0.91	0.085	.895	0.11	.027	5.9	5.62	.636	1.4	.489
1 to 2 yr	0.85	0.130		0.02	.574	3.9	4.39		−0.4	.817
2 to 5 yr	0.87	0.133		0.00	.967	4.4	5.05		0.3	.840
5 to 10 yr	0.85	0.158		−0.01	.774	4.7	4.79		0.5	.717
>10 yr	0.86	0.140		Reference		4.2	4.74		Reference	
Category of brain tumor										
Benign	0.93	0.107	.011	0.02	.493	3.2	4.82	0.426	−0.9	.545
Malignant	0.85	0.134		Reference		4.7	4.86		Reference	
Type of brain tumor										
Metastatic brain tumor	0.86	0.151	.679			5.0	6.54	.467		
Malignant lymphoma	0.90	0.128				4.8	8.22			
Glioblastoma	0.84	0.152				5.5	5.14			
Grade 3 glioma	0.85	0.124				4.0	3.43			
Grade 2 glioma	0.87	0.111				4.8	5.13			
Pituitary tumor	1.00	0.000				7.5	10.60			
Schwannoma	0.90	0.142				7.5	7.78			
Meningioma	0.90	0.130				0.9	1.46			
Other benign brain tumors	0.97	0.065				1.8	2.87			
Other malignant brain tumors	0.88	0.167				2.4	3.36			
Stage of treatment										
Cured (under observation only)	0.91	0.113	<.001	Reference		2.9	3.61	.015	Reference	
Stable (under observation only)	0.88	0.118	<.001	−0.03	.262	5.5	5.65	.013	2.2	.047
Stable (under treatment)	0.81	0.144		−0.11	.005	4.3	3.91		1.4	.361
Recurrence/Progression	0.78	0.175		−0.11	.011	7.2	6.10		3.1	.078
Experience with surgical resection of brain tumor										
Experienced	0.86	0.135	.114	−0.03	.433	4.52	4.76	.861	0.3	.866
Not experienced	0.92	0.116		Reference		4.29	5.37		Reference	
Experience with radiation treatment of brain tumor										
Past experience	0.85	0.139	.129	0.02	.549	4.5	4.39	.908	0.8	.468
Undergoing	0.79	0.163		−0.02	.768	5.3	3.61		1.6	.534
Not experienced	0.89	0.118		Reference		4.4	5.63		Reference	
Experience with chemotherapy for brin tumor										
Past experience	0.85	0.140	.022	−0.02	.621	4.2	4.65	.728	−0.9	.465
Undergoing	0.82	0.138		0.03	.593	5.0	4.27		−2.3	.258
Not experienced	0.90	0.112		Reference		4.7	5.41			
Commuting time for hospital visits										
<30 min	0.87	0.110	.794	Reference		4.2	5.54	.678	Reference	
30 to 60 mi	0.87	0.127	.550	−0.02	.525	4.8	4.50	.707	0.7	.595
1 to 2	0.85	0.146		−0.02	.444	4.8	4.96		0.6	.650
>2	0.86	0.149		0.00	.992	3.1	3.90		−2.0	.257

K6, Kessler Psychological Distress Scale; SD, standard deviation; B, non-standardized parial regression coefficient.

^*^Missing answers and answers of “unknown” were excluded.

^**^ANOVA or Student *t*-test (when 2 rows are listed, the second row represents *P* for trend.).

^***^Multivariable regression analysis with multiple imputation for missing and “unknown”.

In family members, low EQ-5D-5L scores were associated with severe financial hardship and having a family member with tumor recurrence/progression (Table 3). High K6 scores in family members were associated with financial hardship, having a family member with tumor recurrence/progression, and longer commuting times for hospital visits. The results of the full and trimmed models were closely similar, again indicating that the findings were robust(Supplementary Material 2-2). The multivariable analysis among only family members with glioma patients also indicated robustness.

**Table 3.** Factors Associated With Health-Related Quality of Life and Psychological Distress Among Family Members of Patients With Brain Tumors (*N* = 103)

	EQ-5D-5L					K6				
	Discription^*^			Full model^***^		Discription^*^			Full model^***^	
	Mean	SD	*P* ^**^	B	*P*	Mean	SD	*P* ^**^	B	*P*
Intercept				0.79	<.001				9.3	.174
Age										
20s	0.83	NA	.951	Reference		9.0	NA	.105	Reference	
30s	0.87	0.122	.466	0.23	.100	9.9	5.92	.013	−6.6	.311
40s	0.88	0.113		0.21	.110	8.7	7.00		−6.9	.268
50s	0.88	0.119		0.19	.152	5.4	5.17		−8.5	.174
60s	0.87	0.117		0.17	.197	5.1	4.95		−8.8	.163
70s	0.91	0.084		0.19	.167	5.7	6.59		−6.5	.307
Patient age										
00s	0.75	0.105	.657			15.5	0.71	.329		
10s	0.84	0.184	.207			10.4	7.70	.334		
20s	0.87	0.103				5.6	4.63			
30s	0.84	0.166				5.0	6.38			
40s	0.91	0.090				5.4	7.40			
50s	0.86	0.137				7.6	6.13			
60s	0.89	0.107				5.9	5.34			
70s	0.90	0.090				5.9	5.44			
80s	0.85	0.060				8.3	6.02			
Gender										
Male	0.90	0.098	.244	0.02	.475	5.1	4.77	.100	−1.2	.403
Female	0.87	0.117		Reference		7.2	6.31		Reference	
Patient Gender										
Male	0.88	0.109	.454			6.8	6.03	.817		
Female	0.87	0.116				6.2	5.89			
Residential Area of Patient										
Kanto or Further North	0.89	0.115	.191			5.5	5.79	.012		
Chubu or Further West	0.86	0.105				8.6	5.73			
Financial security										
High financial security	0.90	NA	.002	−0.03	.826	2.0	NA	<.001	−0.4	.942
Moderate financial security	0.92	0.078	<.001	0.03	.479	3.7	4.46	<.001	−1.0	.637
Average financial security	0.90	0.100		Reference		4.3	4.70		Reference	
Mild financial hardship	0.87	0.111		−0.02	.465	8.8	5.34		3.5	.018
Considerable financial hardship	0.75	0.127		−0.08	.105	13.6	6.79		7.9	.001
Relationship to the patient										
Spouse/Partner	0.89	0.109	.559	Reference		6.0	6.20	.612	Reference	
Parent	0.86	0.134		−0.03	.475	7.5	6.17		−0.3	.892
Child	0.88	0.085		−0.04	.377	7.9	4.87		0.9	.657
Sibling	0.83	0.117		−0.01	.809	6.2	2.95		−0.7	.796
Cohabiting status										
Living with the patient	0.88	0.117	.412	−0.06	.176	6.7	6.19	.579	1.0	.616
Not living with the patient	0.90	0.077		Reference		5.7	4.18		Reference	
Caregiving status										
No	0.89	0.096	.078	Reference		5.7	5.91	.042	Reference	
Yes	0.85	0.133		0.00	.963	8.1	5.70		1.5	.273
Working status										
Working	0.88	0.110	.009	Reference		6.7	5.80	.300	Reference	
On leave	0.71	0.201		−0.04	.630	11.5	8.81		−1.8	.607
Unemployed and seeking work	0.72	NA		0.01	.952	12.0	NA		−0.4	.950
Homemaker	0.90	0.096		0.03	.434	5.8	5.42		−1.8	.275
Not working	0.90	0.064		0.02	.661	5.0	6.49		−0.1	.947
Consultation with others (multiple answers allowed)										
Family/Relatives	0.88	0.105	.952			6.4	5.93	.473		
Peers affected by brain tumors	0.92	0.095	.393			5.5	6.81	.713		
Acquaintances or friends	0.86	0.103	.351			7.5	6.03	.356		
Medical or welfare professionals	0.84	0.090	.079			6.9	4.46	.772		
Public consultation services	0.79	0.087	.102			9.0	6.16	.408		
Private organizations (eg, patient groups)	0.71	NA	.129			8.0	NA	.811		
Time since diagnosis of brain tumor										
<6 mo	0.91	0.111	.021	0.02	.763	8.8	7.20	.043	2.3	.422
6 mo to 1 yr	0.89	0.101	.680	0.01	.837	8.3	7.15	.002	2.3	.339
1 to 2 yr	0.79	0.129		−0.05	.292	9.0	6.52		1.2	.611
2 to 5 yr	0.91	0.093		0.02	.586	5.7	4.71		0.8	.667
5 to 10 yr	0.88	0.122		−0.02	.742	4.7	5.66		−0.1	.971
>10 yr	0.90	0.084		Reference		3.6	2.84		Reference	
Category of brain tumor										
Benign	1.00	0.000	.237	0.12	.233	7.0	9.90	.618	−2.2	.647
Malignant	0.88	0.112		Reference		6.7	5.93		Reference	
Type of brain tumor										
Metastatic brain tumor	0.93	0.117	.437			7.8	4.60	.928		
Malignant lymphoma	0.82	0.119				7.2	6.83			
Glioblastoma	0.89	0.110				6.8	6.15			
Grade 3 glioma	0.86	0.099				6.9	6.16			
Grade 2 glioma	0.86	0.081				5.3	4.95			
Schwannoma	1.00	0.000				7.0	9.90			
Meningioma	0.91	0.121				8.5	3.54			
Other benign brain tumors	0.96	0.077				1.7	2.08			
Other malignant brain tumors	0.85	0.164				7.1	6.76			
Stage of treatment										
Cured (under observation only)	0.91	0.088	.001	Reference	3.7	4.17	<.001	Reference		
Stable (under observation only)	0.90	0.105	.001	−0.02	.634	5.0	4.27	<.001	0.9	.642
Stable (under treatment)	0.88	0.095		−0.02	.706	8.4	6.67		1.3	.531
Recurence/Progression	0.81	0.143		−0.08	.073	10.2	6.58		3.9	.097
Experience with surgical resection of brain tumor										
Experienced	0.88	0.107	.250	0.00	.942	6.2	5.85	.085	−1.0	.643
Not experienced	0.84	0.154		Reference	9.8	6.12		Reference		
Experience with radiation treatment of brain tumor										
Past experience	0.87	0.113	.270	0.03	.529	6.4	5.74	.225	0.5	.812
Undergoing	1.00	0.000		0.15	.189	1.0	0.00		−5.9	.283
Not experienced	0.89	0.104		Reference	8.1	6.93		Reference		
Past experience	0.89	0.101	.180	−0.04	.446	4.8	4.70	.016	−2.0	.431
Undergoing	0.85	0.125		−0.06	.259	8.4	7.10		−0.8	.780
Not experienced	0.91	0.100		Reference	7.5	4.90		Reference		
Patient’s commuting time for hospital visits										
<30 min	0.87	0.101	.851	Reference	5.4	4.89	.713	Reference		
30 to 60 min	0.90	0.132	.827	0.00	.974	6.7	6.33	.264	3.1	.080
1 to 2 h	0.88	0.101		−0.01	.812	6.9	6.40		2.3	.159
>2 h	0.87	0.132		−0.04	0.448	7.6	5.93		3.5	.161

K6, Kessler Psychological Distress Scale; SD, standard deviation; B, non-standardized parial regression coefficient.

^*^Missing answers and answers of “unknown” were excluded.

^**^ANOVA or Student *t*-test (when 2 rows are listed, the second row represents *P* for trend.).

^***^Multivariable regression analysis with multiple imputation for missing and “unknown”.

Since financial hardship was an important factor, we visualized its distribution by time since diagnosis (Supplementary Material 3).

### Visualizing the Effect of Time since Diagnosis on Families Affected by Patients’ Brain Tumors

To identify specific time periods during which families (both patients and family members) should be prioritized for focused care, the findings related to time since brain tumor diagnosis (Tables 2 and 3, and Supplementary material 2) were visualized as graphs. Based on the results above, EQ-5D-5L was considered the primary quality-of-life indicator for patients, while K6 was considered more representative for family members. Consequently, we plotted the trajectory of patient EQ-5D-5L scores and family member K6 scores over time. To ensure a valid comparison between patients and family members, we also displayed results from multivariable regression analyses of the matched subsample (*n* = 39). For comparability between EQ-5D-5L and K6, the Y-axis for K6 was inverted, so higher values indicated better QOL.

**Figure 2.**
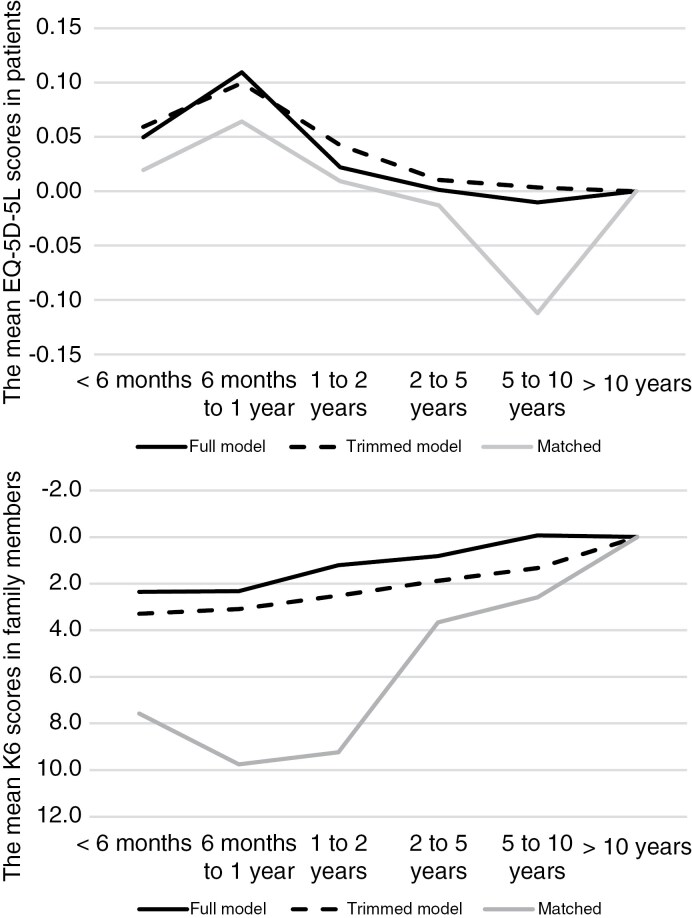
Quality of life indicators in patients and family members by duration from brain tumor diagnosis. K6, Kessler Psychological Distress Scale. Mean scores are displayed as a difference from “>10 years” due to the lack of baseline (pre-diagnosis) data, with “>10 years” treated as the reference value in regression analyses.

The average EQ-5D-5L scores for patients between 6 months and 1 year after diagnosis were higher than those from the period prior to 6 months, but showed a clear decline thereafter (Figure 2). In contrast, the average K6 scores for family members appeared to be stable or showed a slow improvement in psychological distress over time. The trajectories of patients and family members were not synchronized. Results from the matched subsample further supported the existence of a time lag in trajectories. To note, a dendrogram used in the matching procedure is shown in Supplementary Material 4 and may help illustrate how certain background similarities aligned between patients and family members.

## Discussion

In this study of HRQL and psychological distress and associated factors in patients with brain tumors and their family members, we found that HRQL in patients was significantly impacted by their brain tumors. The degree of impact was associated with sociodemographic and tumor-related characteristics. Similarly, psychological distress in family members was exacerbated by the patient’s brain tumor and was influenced by their own sociodemographic and tumor-related characteristics, as well as the patient’s geographical context. The relatively large sample size enhanced the robustness of these findings, which provide valuable insights into when and how health and community specialists can effectively support the well-being of families affected by brain tumors.

The participants demonstrated a wide range of characteristics, including a notable proportion of working individuals. The importance of balancing cancer treatment and work has recently been highlighted.^23^ In particular, HRQL is lower among homemaking patients, which in turn indicates that quitting work to become a homemaker does not necessarily mean that their situation is made easier. For many patients, the workplace may act as a source of social support, akin to acquaintances or friends. Conversely, homemakers may face additional challenges due to the multitasking nature of their role, which includes managing household responsibilities alongside self-care (by patients) or caregiving (by family members). Furthermore, balancing work with multiple responsibilities—such as caregiving for brain tumor patients, caring for other dependents (eg, children), and maintaining one’s own mental health—should involve greater concern. Some brain tumor patients were also responsible for caregiving for other family members, such as older adults. The division of household tasks and responsibilities is further complicated by the ongoing reduction in household size, highlighting the need for family support systems that address these complexities.

Our finding of impaired QOL in patients, particularly HRQL, is consistent with previous studies.^5,6^ EQ-5D-5L proved to be an effective indicator of patient difficulties. However, the finding of low HRQL among homemakers with brain tumors is novel and to our knowledge has not been previously reported. One possible explanation is the cognitive challenges faced by patients with brain tumors, such as higher brain dysfunction, which may make the multitasking demands of homemaking particularly difficult. Accordingly, we speculate that homemakers may benefit from support systems similar to workplace accommodations, such as task coordination assistance.

Our finding of impaired QOL of family members was also significant. Notably, levels were comparable to those of the patients themselves. Sun et al.^15^ reported a median K6 score of 3 (IQR: 0–7) among caregivers of cancer patients based on a national survey in Japan. In contrast, caregivers of brain tumor patients in this study exhibited higher psychological distress, suggesting that caregiving for brain tumors poses unique challenges. A brain tumor can be considered a type of brain injury, causing cognitive and personality changes to be managed among families.^24^ These challenges include managing cognitive and personality changes in patients, the prolonged burden of caregiving during end-of-life stages, and anxiety over the lack of standard treatment protocols.

A novel finding of this study is the impact of patients’ commuting time for hospital visits on their family members’ psychological distress. A previous study of parents of children undergoing solid organ transplantation reported worse mental HRQL in parents with longer commuting times to follow-up hospitals.^25^ Similarly, caregivers of brain tumor patients often accompany patients to hospital visits, leading to increased burden. In addition, long distances may exacerbate anxiety by creating concerns about delayed access to immediate assistance in cases of emergency. Further support by mobility services would be required.

Financial security was a significant factor affecting the QOL of both patients and family members. On adjustment for financial security by multivariable analysis, the effect of working status mostly disappeared, except with regard to patient homemaking and HRQL, as discussed above. Regarding socioeconomic status, previous studies have shown that education level in adults with brain tumors is associated with the functional living index-cancer score,^26^ which measures different dimensions of QOL.^4^ Similarly, low income among the parents of children with brain tumors has been linked to reduced HRQL.^11^ Our study produced similar findings. An important outcome of our study is that financial hardship was associated with poorer QOL for both patients and family members, whereas high or moderate financial security did not necessarily correspond to better QOL. This suggests that the relationship between financial status and QOL is not linear. Accordingly, financial support should be specifically targeted at high-risk subgroups experiencing financial hardship and continue until they can feel a sense of normalcy.

This study also evaluated time since brain tumor diagnosis. HRQL was highest in patients surveyed between 6 months and 1 year after diagnosis, after which HRQL appeared to decline. Conversely, the psychological distress of family members appeared to slightly increase during the same period. These trends were consistent regardless of tumor treatment stage or whether the matched or original sample was analyzed. This finding suggests a difference in the timing of when patients and family members specifically require additional support. This is surprising, given that both groups share a common experience within the same family. One possible explanation for this time lag is that families (both patients and family members) support each other during the caregiving process. The family impact of a brain tumor may manifest most clearly in the family member (including the patient) who is most burdened at a particular time, with this burden shifting between family members as circumstances change. This is consistent with the notion that families develop adaptive systems and regulate interactions, and thereby influence the distribution of caregiving burdens.^27^ Most participants in this study, both patients and family members, sought consultation from family or relatives when needed. The families in this study therefore, appear to resolve issues internally within the family and rarely seek external support, such as peer support groups. This characteristic of the sample may partly explain the observed time lag between patients and family members.

Several limitations of this study should be noted. First, it included a higher proportion of participants with malignant brain tumors, which may reflect the characteristics of the recruitment institutions. Epidemiologically, malignant brain tumors are more prevalent in males,^1^ and the study included more male patients and female family caregivers, often as spouses. A more comprehensive understanding of differences between benign and malignant cancers will require further research with a larger number of patients with benign brain tumors and affected family members. The sampling strategy also introduces other limitations. For example, only a small number of caregivers of pediatric patients were included. These factors may have introduced a degree of self-selection bias.

Second, while we conducted paired analyses using probability matching, this method has not been validated, and it was not possible to confirm whether the matched pairs truly belonged to the same family. The approach was applied post hoc as a sensitivity analysis and relied on similarity in background characteristics rather than shared identifiers. While the mutual closest-match criterion and the exclusion of implausible pairings (eg, patient–patient or caregiver–caregiver) may have increased the likelihood of true matches, errors in self-reported data and chance similarities remain possible. A dendrogram illustrating the matching structure is provided in Supplementary Material 4 to enhance transparency. These results should be interpreted with caution, and we emphasize that the primary analyses were conducted using unmatched group-level data.

Third, the cross-sectional nature of the evaluation warrants mentions as a limitation. Although we examined differences based on time since brain tumor diagnosis and discussed the trend inconsistency between patients and family members, these findings do not account for each individual’s trajectory. Further research is needed to clarify these dynamics. One approach would be to conduct longitudinal studies that can reveal the trajectories of both patients and family members, as well as the temporal order of changes in HRQL and psychological distress. Another approach would be to use retrospective questions that explore the temporal relationship between disease progression and life events, such as changes in employment status or caregiving roles. In this study, we identified factors such as financial hardship or being a homemaker as associated with lower HRQL. However, it remains unclear whether these statuses existed prior to the disease onset or were a consequence of the illness. Future studies that address these temporal relationships will allow for a more comprehensive understanding of how brain tumors impact both patients and their family members over time.

In conclusion, this study evaluated levels of HRQL and psychological distress in patients with brain tumors and their family members, and identified key factors associated with these outcomes. The results showed that HRQL in patients was significantly affected by financial hardship, role as a homemaker, longer duration since diagnosis, and ongoing treatment or tumor recurrence/progression. In contrast, psychological distress among family members was influenced by financial hardship, tumor recurrence/progression in the patient, and longer commuting time for hospital visits. Further research is required to guide the development of community care aimed at supporting families as a whole, rather than focusing solely on patients or individual family members.

## Supplementary Material

vdaf098_suppl_Supplementary_Material_S1

vdaf098_suppl_Supplementary_Material_S2

vdaf098_suppl_Supplementary_Material_S3

vdaf098_suppl_Supplementary_Material_S4

## Data Availability

Owing to the nature of this research, the participants of this study did not agree to the public sharing of their data, and supporting data are not available.
