# Supplementary material for: Health-related quality of life and psychological distress in patients with brain tumors and their families: A cross-sectional web survey
Source: Neurooncol Adv. 2025 May 18;7(1):vdaf098. doi: 10.1093/noajnl/vdaf098 (PMC12202142; doi:10.1093/noajnl/vdaf098)
Supplement: vdaf098_suppl_Supplementary_Material_S1 [file vdaf098_suppl_supplementary_material_s1.docx]

Supplemental material 1. Demographic questionnaire (translated into English)

| Content | Question | Response Options |
| --- | --- | --- |
| Age | Please tell us your age.† | 00s/10s/20s/30s/40s/50s/60s/70s/80s/90s |
| Gender | What is your gender? † | Male/Female/Others |
| Residential Area | Please indicate the prefecture where you currently reside.* | (The 47 prefectures in Japan) |
| Financial Security | How do you feel about your overall current living situation? | High financial security / Moderate financial security / Average financial security / Mild financial hardship / Considerable financial hardship |
| Relationship to the Patient | From the patient's perspective, what is your relationship to them?‡ | Spouse or partner / Parent / Child / Sibling / Others |
| Cohabiting Status | Do you currently live with the patient?‡ | Living with the patient (including when they are temporarily hospitalized) / Not living with the patient |
| Caregiving status | How many people in your household require assistance or supervision? Please select all applicable options from the list below. | Children who have not yet entered elementary school / Elderly people who require support or long-term care / Others who require assistance or supervision due to a disability or other reasons |
| Working Status | Please indicate your employment status during the past week. | Working / On leave / Unemployed and seeking work / Student / Homemaker / Not working |
| Consultation with others | Please select all of the people you are connected with from the following list, who you can rely on in times of need.  (multiple answers allowed) | Family or relatives / Friends or acquaintances in a similar situation related to brain tumors / Friends, acquaintances, or associates from school, workplace, or local community / Medical professionals or welfare service providers / Professionals or staff from public organizations / Professionals or staff from private organizations |
| Time since diagnosis of brain tumor | How long has it been since you were diagnosed with a brain tumor?* | < 6 months / 6 month to 1 year / 1 to 2 years / 2 to 5 years / 5 to 10 years / > 10 years |
| Category of brain tumor | Please indicate whether your brain tumor is benign or malignant.* | Malignant brain tumor / Benign brain tumor/ Unknown |
| Type of brain tumor | Which of the following types of brain tumors applies to you?* | Grade 2 glioma / Grade 3 glioma / Glioblastoma / Malignant lymphoma / Metastatic brain tumor / Other malignant brain tumors / Meningioma / Pituitary tumor / Schwannoma / Other benign brain tumors / Unknown |
| Stage of treatment | Please indicate the current status of your brain tumor.* | Cured (under observation only) / Stable (under observation only) / Stable (under treatment) / Recurrence or Progression |
| Experience with surgical resection of brain tumor | Have you ever undergone surgery for a brain tumor?* | Yes/ No |
| Experience with radiation treatment of brain tumor | Have you ever undergone radiation therapy?* | Undergoing / Yes / No |
| Experience with chemotherapy for brain tumor | Have you ever undergone chemotherapy?* | Undergoing / Yes / No |
| Commuting time for hospital visits | Please indicate the time required to commute to the hospital.* | < 30 minutes / 30 to 60 minutes / 1 to 2 hours / > 2 hours |

* Wording in questions directed to family members has been slightly modified to refer to the patient rather than the family caregiver themselves.

† For family members, two versions of this question were asked: one about themselves (your), and one about their family member with a brain tumor.

‡ This question was asked only to family members.
