# Supplementary material for: Health-related quality of life and psychological distress in patients with brain tumors and their families: A cross-sectional web survey
Source: Neurooncol Adv. 2025 May 18;7(1):vdaf098. doi: 10.1093/noajnl/vdaf098 (PMC12202142; doi:10.1093/noajnl/vdaf098)
Supplement: vdaf098_suppl_Supplementary_Material_S3 [file vdaf098_suppl_supplementary_material_s3.pptx]

## Slide 1
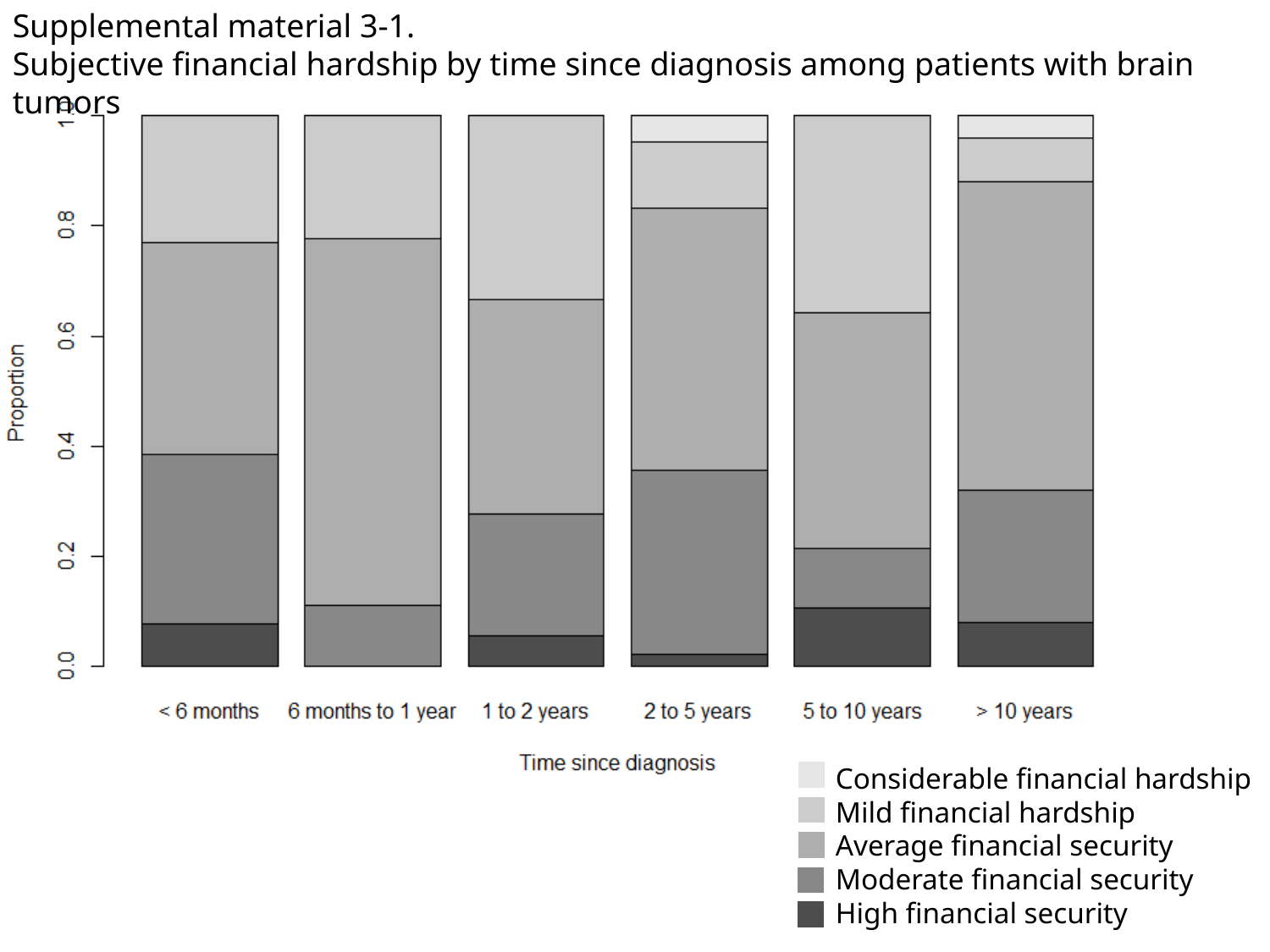

Supplemental material 3-1.
Subjective financial hardship by time since diagnosis among patients with brain tumors
Considerable financial hardship
Mild financial hardship
Average financial security
Moderate financial security
High financial security

## Slide 2
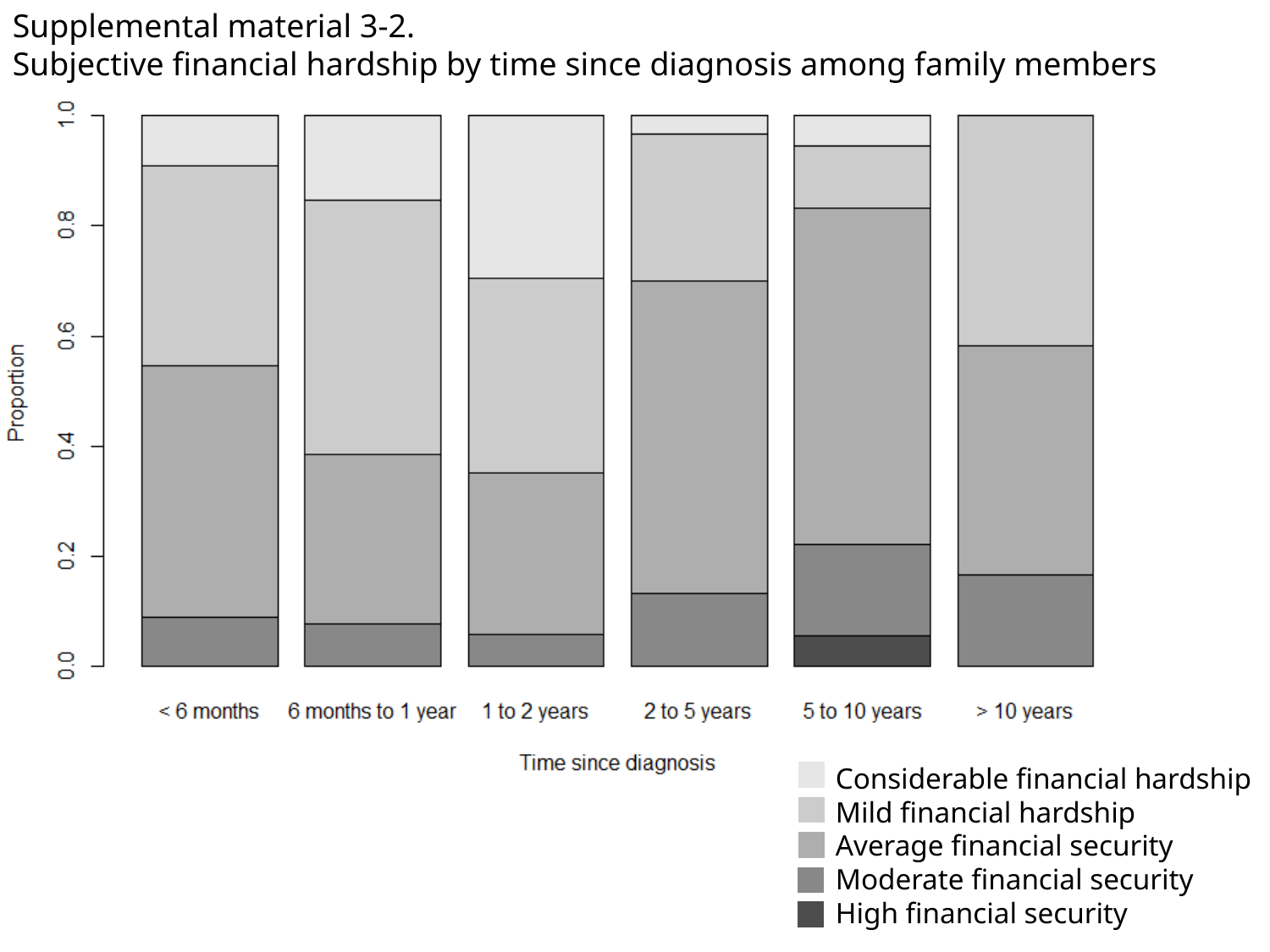

Supplemental material 3-2.
Subjective financial hardship by time since diagnosis among family members
Considerable financial hardship
Mild financial hardship
Average financial security
Moderate financial security
High financial security
